# Electronic Health

**DOI:** 10.1155/2009/308710

**Published:** 2009-03-30

**Authors:** Hui Chen, Arnauld Nicogossian, Silas Olsson, Azhar Rafiq, Max E. Stachura, Mamoru Watanabe, Pamela Whitten, Yang Xiao

**Affiliations:** ^1^Department of Mathematics and Computer Science, Virginia State University, P.O. Box 9068, Petersburg, VA 23806, USA; ^2^Center for the Study of International Medical Policies and Practices, School of Public Policy, George Mason University, Fairfax, VA 22030, USA; ^3^AAL International Association, Bischoffsheimlaan 25, 1000 Brussels, Belgium; ^4^Center for Human Simulation and Patient Safety, School of Medicine, Virginia Commonwealth University, P.O. Box 980480, Richmond, VA 23298, USA; ^5^Center for Telehealth, Medical College of Georgia, Augusta, GA 30912, USA; ^6^Health Innovation and Information Technology Centre, Faculty of Medicine, University of Calgary, Calgary, AB, Canada T2N 1N4; ^7^College of Communication Arts and Sciences, Michigan State University, MI 48824, USA; ^8^Department of Computer Science, University of Alabama, P.O. Box 870290, Tuscaloosa, AL 35487-0290, USA

Computing and networking techniques and technologies are gradually penetrating into every aspect of health care and medical practice. Many believe that important benefits can be achieved with the marriage of these two areas, including the delivery of high-quality health care at lower cost. Electronic health (e-Health) has become a very important area of focus and activity in multiple domains, such as health promotion, health care and maintenance, public health, medical science, health service, data management, image processing, telecommunication, wireless network, and operational research. The purpose of the special issue is to provide a high quality exposure to advances and experiences in e-Health related topics and to stir interest in advancing e-Health to the next level.

The call for papers for this special issue attracted numerous exciting responses from the research community. Submissions covered diverse areas of interests. Unfortunately, space limitations allowed only nine papers to be selected for inclusion in this special issue, after extensive peer review. In the following paragraphs, we briefly introduce the nine accepted papers.

Health information technology plays an important role in supporting decision making, health care delivery, and management of health services. Many sociotechnical factors affect physicians' adoption and implementation of health information systems. Having conducted semistructured interviews with 26 physicians from nine medical clinics in Alberta, Canada, D. A. Ludwick and John Doucette present a discussion of the barriers to implementing health information systems in a fee-for-service environment in their paper entitled “Primary care physicians' experience with Electronic Medical Records.”

A potential obstacle impairing the adoption of health information systems might be simply that developed systems do not meet user needs. A. Shaban-Nejad et al. examined system requirement management in their paper “Managing Requirement Volatility in an Ontology-Driven Clinical Laboratory Information Management System (LIMS) Using Category Theory.”

Security and privacy are important requirements for health information systems. For example, patients' privacy is protected by law in many countries. In Anastasios Fragopoulos et al. paper entitled “Context Aware Security for Pervasive Healthcare Architectures Utilizing MPEG-21 IPMP Components,” MPEG-21 intellectual property management and protection components are used to achieve protection of transmitted medical information and enhance patient privacy.

Abdulmutalib Masaud-Wahaishi and Hamada Ghenniwa present an information brokering architecture that supports privacy-based information gathering in healthcare in their paper entitled “Agent-Oriented Privacy-Based Information Brokering Architecture for Healthcare Environments.” In their proposed architecture, a brokering service is modeled as an agent with a specific architecture and interaction protocol appropriate to serve various requests.

Agent technology is gaining momentum in modeling and developing complex software systems. The next paper discusses major features and benefits of an agent-based approach to enhance a hospital laboratory legacy information system. This paper entitled “Enhancing E-Health Information Systems with Agent Technology” is contributed by Minh Tuan Nguyen et al.

The next two papers heavily focus on roles of communications and networking aspects in e-Health systems. In their paper entitled “An adaptive Source-Channel Coding with Feedback for Progressive Transmission of Medical Images,” Jen-Lung Lo et al. studied source-channel coding problem with the consideration of characteristics of medical images.

IEEE 802.15.4 standard specifies the physical layer and the media access control layer for wireless personal area networks with low data rate and low power consumption. It forms the basis for many body sensor networks. IEEE 802.11 standards are the most widely adopted wireless local area network (LAN) standards. Jelena Misic and Xuemin (Sherman) Shen modeled an interconnected network consisting of IEEE 802.15.4 body sensor networks and IEEE 802.11 wireless LANs. Their paper is entitled “Delay Analysis of GTS Bridging between IEEE 802.15.4 and IEEE 802.11 Networks for Healthcare Applications.”

The last two papers provide us with the examples of e-Health systems at work. C. Quantin et al. show that it is possible to set up a continuous and exhaustive recording system for linked perinatal data to assess the quality of care on a regional scale in their paper entitled “Using discharge abstracts to evaluate a regional perinatal network: assessment of the linkage procedure of anonymous data—using discharge abstracts to evaluate a regional perinatal network.”

Jean-François Lesesve and Richard Garand demonstrate one of the first approaches to the use of telehematology for the quality control of diagnosis using the GOELAMS Chronic Lymphocytic Leukaemia 98 trial. Their paper “Evaluation of a Telemedicine System for the Transmission of Morpho/Immunological Data Aiming at the Inclusion of Patients in a Therapeutic Trial” details their experience and the benefits arising from the use of telehematology in clinical practice.

In summary, this special issue includes papers that span diverse areas of interests including system development, security and privacy, actual effect of e-Health in practice, as well as technological foundations. It was our honor to receive submissions from many authors. Without our unselfish reviewers, who provided us with extensive and constructive reviews, it would not be possible to run a special issue of such broad scope. Last, but not least, the publishing staff have worked diligently with us on this special issue. We are, therefore, indebted to all the authors, the reviewers, and publishing staff. We would like to express our sincere gratitude to them all.

*Hui Chen* *Hui Chen* *Arnauld Nicogossian* *Arnauld Nicogossian* *Silas Olsson* *Silas Olsson* *Azhar Rafiq* *Azhar Rafiq* *Max E. Stachura* *Max E. Stachura* *Mamoru Watanabe* *Mamoru Watanabe* *Pamela Whitten* *Pamela Whitten* *Yang Xiao* *Yang Xiao*

